# Impact of worker emigration on HIV epidemics in labour export areas: a molecular epidemiology investigation in Guangyuan, China

**DOI:** 10.1038/s41598-018-33996-6

**Published:** 2018-10-30

**Authors:** Ling Su, Shu Liang, Xueqin Hou, Ping Zhong, Dongbing Wei, Yu Fu, Li Ye, Li Xiong, Yali Zeng, Ying Hu, Hong Yang, Bo Wu, Linglin Zhang, Xiaoshan Li

**Affiliations:** 1 Center for AIDS/STD Control and Prevention, Sichuan Provincial Center for Disease Control and Prevention, Sichuan, China; 2 Center for AIDS/STD Control and Prevention, Guangyuan Municipal Center for Disease Control and Prevention, Gunagyuan, China; 3 Department of AIDS and STD, Shanghai Municipal Center for Disease Control and Prevention, Shanghai Municipal Institutes for Preventive Medicine, Shanghai, China; 4 Department of Lung Transplant Center, Affiliated Wuxi People’s Hospital of Nanjing medical University, Wuxi, Jiangsu China

## Abstract

We aimed to investigate the molecular epidemic characteristics and viral transmission patterns of HIV-1 in a typical labor export area, Guangyuan city, China. Based on conducting phylogenetic trees and molecular transmission networks, a phylogenetic analysis was performed on HIV-1 *pol* sequences obtained from 211 migrant-history workers, 83 non-migrant-history individuals, and 21 migrant-history unknown individuals between January, 2012 and February, 2017 in Guangyuan city. Phylogenetic analysis revealed that CRF07_BC (48.3%, n = 152) and CRF01_AE (33.3%, n = 105) were the dominant strains in Guangyuan city, and circulated by multiple lineages with various epidemic characteristics. Geographic network analysis showed that Guangyuan city-related sequences with 20.3% CRF07_BC and 28.3% CRF01_AE were linked to that of other provinces, compared to that with 1.7% CRF07_BC and 5.0% CRF01_AE in cities of Sichuan. Molecular transmission network analysis further illustrated that migrant-history workers linked more sequences from other provinces than non-migrant-history individuals in both CRF07_BC (29.3% versus 0.0%, *P* = 0.013) and CRF01_AE (40.5% versus 10.0%, *P* = 0.001) networks. Our results highlighted that migrant-history workers in recent year played a vital role in fueling HIV-1 epidemic in Guangyuan city. Molecular transmission network analysis could be a useful approach for disclosing the transmission mechanism of HIV, which should be used in prevention and intervention efforts.

## Introduction

By the end of December 2017, approximately 758,610 people were living with HIV-1 in China, with an estimated 321,233 cases of AIDS. Among the 134,512 newly diagnosed HIV-1 infections in 2017, 69.4% was attributed to heterosexual and 25.3% to homosexual transmission^1^. One of the current challenges was that China faced the rapid increase of HIV-1 prevalence among floating population and lacking effective intervention strategies for this group^2,3^. With the rapid urbanization of China, outgoing migrant-workers had become the main component of the floating population in China, who worked outside the place where the household registrations were located^4^. The migrant-workers accounting for a large proportion of HIV-1 epidemic in China had caused the diversity of circulating HIV-1 and could further local fuel epidemics^5,6^. Although several molecular epidemiological investigations conducted in Shanghai, Beijing, Shenzhen, and others indicated that migrant-workers had a significant influence on the HIV-1 epidemics in coastal or metropolis^2,7,8^, barely researches focused on the condition of rural areas where export labors.

**Figure 1:**
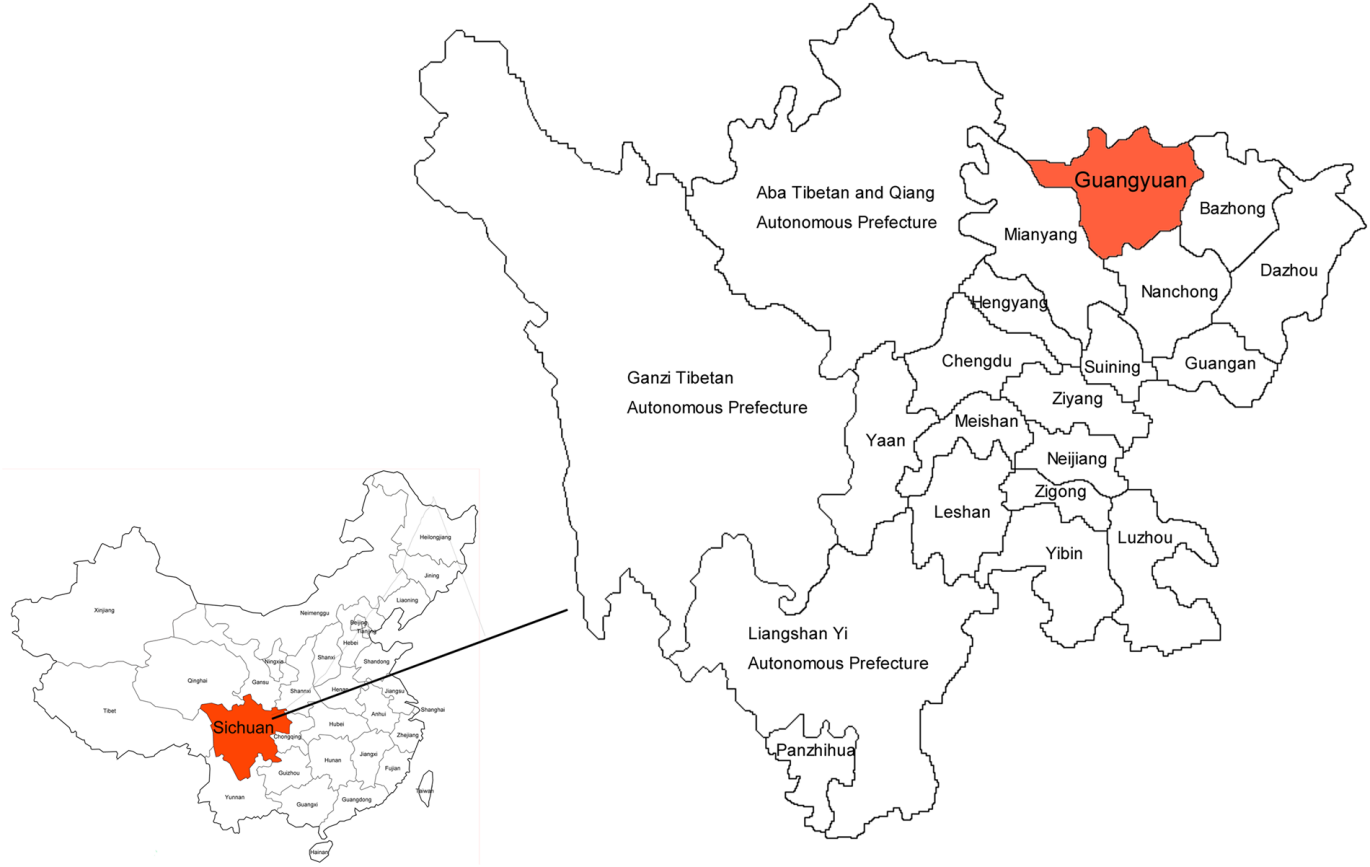
The geographical location of Guangyuan in China.

The newly diagnosed cases of HIV-1 in Sichuan province ranked first in China in 2017, and the prevalence of HIV-1 varied widely in regions^1^. The presence of relatively poor and developing towns and counties had made Sichuan become an important labor-exporting province, with a rapid increase in the prevalence of HIV-1. Guangyuan city, located in northern Sichuan province in western China (Fig. 1), was a mountainous agricultural city with 3.05 million registered populations currently. It was also renowned for its large output of labors in Sichuan. Every year, approximately one million migrant-workers were far away from their homes to seek better employment opportunities, including 21% inside and 54% outside their home province. Above 50% of them had become major migrant labors in Yangtze (mainly including Jiangsu and Shanghai), Zhujiang River delta areas (mainly located in the southeast of Guangdong), and the Bohai Rim (mainly including Beijing and Tianjin)^9,10^. Since the first HIV-1 case was identified in 1996 in Guangyuan city, the cumulative number of reported HIV-1-infected persons had reached 978 in 2017 (from a report of Guanyuan Municipal Center for Disease Control and Prevention[CDC]). In recent six years, the number of newly diagnosed HIV-1 infections increased from 81 in 2012 to 196 in 2017, showing a serious situation in HIV-1 prevalence. Therefore, we characterized HIV-1 genetic diversity and transmission network in this labor-exporting representative city, Guangyuan city, and tried to explore a possible mechanism for controlling the spread of HIV-1 in this kind of areas.

## Methods

### Study participants

From all local voluntary counseling and testing sites (VCT), sentinel surveillance sites, and medical institutions, a total of 651 HIV-1 patients were newly diagnosed between January 1, 2012 and February 28, 2017 in Guangyuan city. Among them, 66.7% (434) were recruited by the staffs of CDC for participating in the study within the first year after HIV diagnosis. The socio-demographic characteristic indicators included age, gender, level of education, marital status, and risk group were collected by the staffs of CDC simultaneously. All individuals read the content of the study and signed written informed consent. The other individuals could not be recruited for loss of following up (26, 4.0%) or refusal to participate (191,29.3%).

### Sequence data collection

A total of 10 mL whole blood samples were collected from the 434 studied participants. None of the individuals was exposed to antiretroviral treatment (ART) at the time of blood specimen collection. Plasma was separated from the whole blood within two hours after collection and stored at −80 °C until use. Blood plasma samples were subjected to viral RNA extraction and cDNA synthesis as previously described^11^. HIV-1 *pol* sequences (protease 1–99 and reverse transcriptase 1–250 amino acids) was amplified and sequenced. Eventually, a total of 315 HIV-1 *pol* sequences covering 1,060 base pairs (HXB2: 2,253–3,312) were successfully obtained. The sampling rate of all the diagnosed patients in Guangyuan city was 48.4% (315/651). (Supplementary Material 1).

### Migration condition data collection

The 315 individuals with *pol* sequences were further conducted a questionnaire survey after sequence data collection. Migration condition data were collected through face-to-face or telephone interviews conducted by the staffs of CDC. These indicators included whether the subjects had gone out as migrant worker before diagnosed, age of the first going out as migrant worker, occupation of going out as migrant worker, and place of migrant working. 294 questionnaires were collected.

### Identification of HIV-1 subtypes and CRFs

The HIV-1 *pol* sequences of studied samples were aligned with reference sequences of subtypes and circulating recombinant forms (CRFs) (Los Alamos HIV sequence database [LANL], http://www.hiv.lanl.gov/) using the Mega version 7.0 with minor manual adjustments. FastTree 2.3 was used to construct an approximately-maximum-likelihood phylogenetic tree (ML tree) using the GTR + G + I nucleotide substitution model^12^. The phylogenetic tree’s reliability was determined with local support values based on the Shimodaira-Hasegawa (SH) test^13^ and presented using FigTree v1.3.1 (http://beast.bio.ed.ac.uk).

### Identification of HIV-1 epidemic lineages

For further analysis of HIV-1 epidemic lineages of predominant variants circulating in Guangyuan city, a BLAST-based search tool (https://www.hiv.lanl.gov/content/sequence/BASIC_BLAST/basic_blast.html) was used to search against the closely related sequences from locals and nationwide in Sichuan province CDC drug resistance database and LANL database. Two sets of databases, database_CRF07_BC_ with 664 CRF07_BC *pol* sequences and database_CRF01_AE_ with 584 CRF01_AE *pol* sequences were built for both phylogenetic and molecular transmission network analyses. A monophyletic group in ML phylogenetic tree(built as mentioned above) with bootstrap support ≥0.9 was considered as an epidemic lineage.

### Analysis of HIV-1 molecular transmission network

The flow chart of transmission network analysis included four steps: construction of phylogenetic tree, extraction of transmission cluster, identification of minimum patristic genetic distance and visualization of the network^3^. We used Cluster Picker^14^ to extract transmission clusters from the phylogenetic tree, with the intra-cluster maximum pairwise distance <3.0% nucleotide substitutions per site and bootstrap support value ≥95%. The patristic genetic distances of all sequences within the available clusters were calculated in Patristic software (http://www.bioinformatics.org/patristic/manual.html) (Supplementary Material 2). Among all distances, one that minimizes the sum of edge weights (patristic genetic distances) was selected to define the linkages within a cluster^3^. Lastly, the network data were visualized and analyzed using the network software Cytoscape 3.5^15^.

### Statistical analysis

Chi-square test was used to calculate the distributions of demographic information between CRF01_AE and CRF07_BC, and compare the difference of linkages between migrant-history workers and non-migrant-history individuals in transmission networks. A *p-*value less than 0.05 was considered statistically significant. All statistical analyses were performed using SPSS v.20.0 software (IBM Company, New York, USA).

### Ethics statement

The study protocol was reviewed and approved by the Institutional Review Board at the Human Medical Research Ethics Committee of the Sichuan CDC. The objectives and the procedure of the study, and potential risks and benefits of participating in the study were given to potential participants during the recruitment of study subjects. Verbal and written consent procedures were given to the study participants and they had the right to discontinue the survey at any time. All research methods in this study were carried out by the approved guidelines.

## Results

The mean age of the 315 participants was 39.4 ± 14.0 years (range 18–78) and male-to-female ratio was 3.5:1. Of these, 132 were single (41.9%), and 182 (57.8%) received primary education or below. The transmission routes of these subjects included 141 (44.8%) male heterosexuals, 86 (27.3%) men who have sex with men (MSM), 65 (20.6%) female heterosexuals, and 23 (7.3%) other transmission routes. The median CD4+ T cell count was 272.0 (IQR: 138.3~377.5) cells/μl (Table 1).

**Table 1:** Socio-demographic characteristics for subjects from Guangyuan.

Demographic characteristics	Number (%)
**Age (years)**	
≤30	99 (31.4)
31–40	82 (26.0)
41–50	65 (20.7)
51–60	36 (11.4)
≥61	33 (10.5)
**Sex**	
Male	245 (77.8)
Female	70 (22.2)
**Marital Status**	
Single	132 (41.9)
Married	162 (51.4)
Unknown	21 (6.7)
**Years of Education**	
0–6	56 (17.8)
7–9	126 (40.0)
≥10	114 (36.2)
Unknown	19 (6.0)
**Risk group** ^a^	
M-HET	141 (44.8)
F-HET	65 (20.6)
MSM	86 (27.3)
PWID/SU	23 (7.3)
**Sampling year**	
2012	49 (15.6)
2013	49 (15.6)
2014	79 (25.1)
2015	66 (20.9)
2016	54 (17.1)
2017	18 (5.7)
**CD4+ T cell count (cells/μl)**	
<200	103 (32.7)
200–349	107 (34.0)
350–500	65 (20.6)
≥500	35 (11.1)
Unknown	5 (1.6)
**Gone out as migrant worker before diagnosed** ^b^	
Yes	211 (67.0)
No	83 (26.3)
Unknown	21 (6.7)
**Age of the first going out as migrant worker (years)** ^c^	
≤20	63 (29.8)
21–30	48 (22.8)
31–40	19 (9.0)
≥40	21 (9.9)
Unknown	60 (28.5)
**Place of migrant working** ^c^	
Inside Guangyuan but outside the domicile	6 (2.8)
Inside the home province but outside Guangyuan	33 (15.7)
Outside the home province	115 (54.5)
Unknown	57 (27.0)
**Occupation of going out as migrant worker** ^c^	
Worker	78 (37.0)
Waiter	33 (15.6)
Company employee	16 (7.6)
Salesman	14 (6.6)
Driver	4 (1.9)
Others	4 (1.9)
Unknown	62 (29.4)

^a^M-HET, male heterosexuals; F-HET, female heterosexuals; MSM, men who have sex with men; PWID, persons who inject drugs; SU, sexual transmission, unspecified type.

^b^The time of going out as migrant worker above 30 days before diagnosed.

^c^The total number was 211.

### High proportion of the migration in Guangyuan city

A total of 67.0% participants reported that they had the experience of being migrant workers before diagnosed, and the mean age of the first going out as migrant workers was 26.9 ± 11.0 years (rang 15.0–70.0). Most of the subjects who ever went out as migrant workers (migrant-history workers) were outside the home province (54.5%), whereas 15.7% and 2.8% stated “inside the home province but outside Guangyuan city” and “inside Guangyuan city but outside the domicile”. The main occupation of these migrant-history workers was worker (37.0%), followed by waiter (15.6%), company employee (7.6%), salesman (6.6%), driver (1.9%), and others (1.9%) (Table 1).

### Extensive diversity of HIV-1 subtypes and CRFs in Guangyuan city

**Figure 2:**
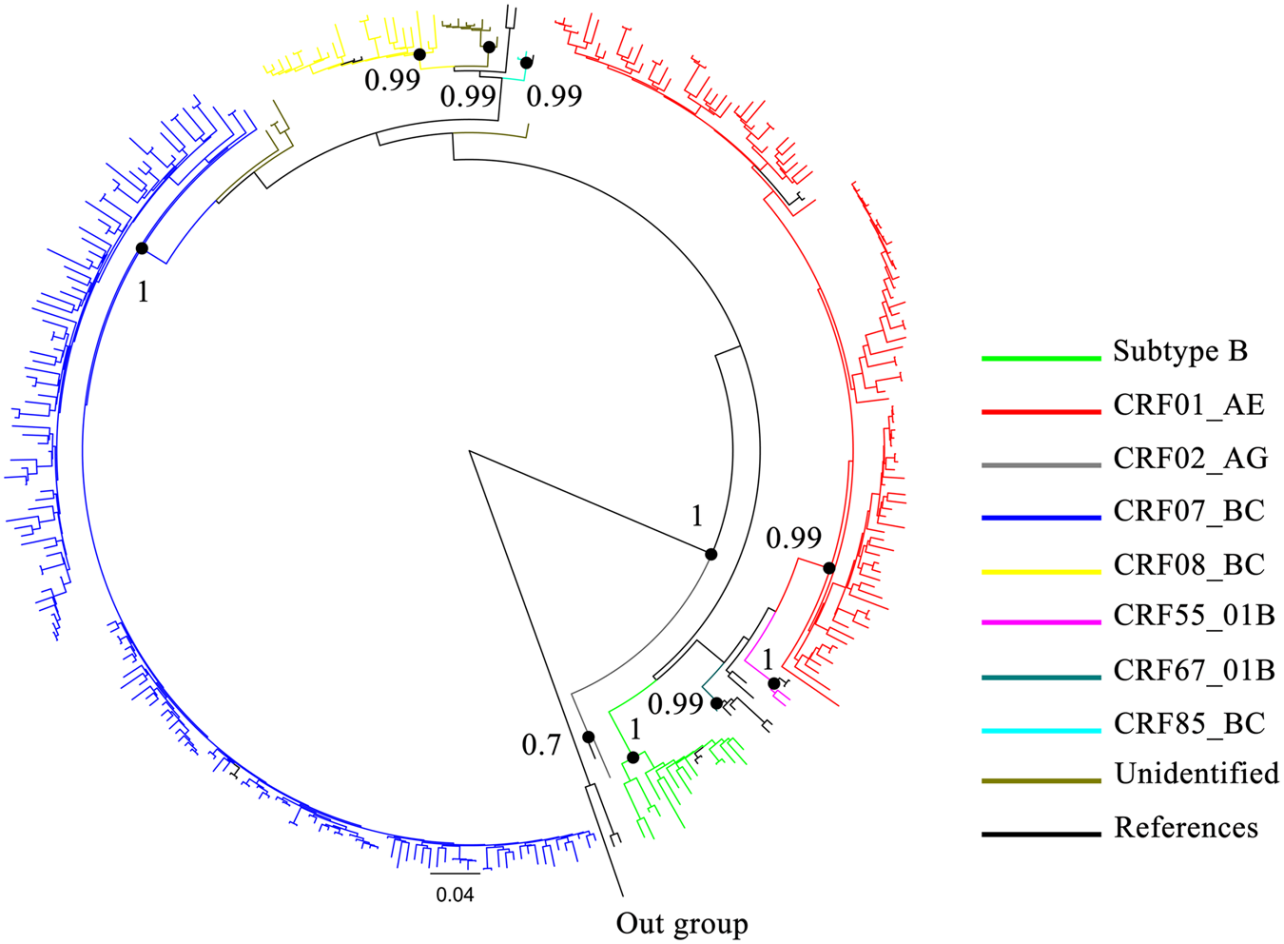
Phylogenetic analysis of *pol* sequences from Guangyuan. The phylogenetic tree was constructed using the approximately-maximum-likelihood method based on the *pol* region (HXB2: 2, 253 to 3, 312 nt) in FastTree 2.3. The nucleotide substitution mode was GTR + G + I. The subtypes and circulating recombinant forms are marked in different colors. The bootstrap valueswere indicated at relevant nodes. HIV-1 group O was chosen as an out-group in the rooted tree. The reference sequences were from the Los Alamos HIV sequence database (http://hiv-web.lanl.gov/content/index).

Phylogenetic analysis based on 315 *pol* sequences revealed the presence of one subtype and seven CRFs in Guangyuan city (Fig. 2). Two predominant strains were found to be CRF07_BC (48.3%, n = 152) and CRF01_AE (33.3%, n = 105), followed by CRF08_BC (7.0%, n = 22), subtype B (5.1%, n = 16), CRF55_01B (0.6%, n = 2), CRF85_BC (0.6%, n = 2), CRF02_AG (0.3%, n = 1) and CRF67_01B (0.3%, n = 1). In addition, unique recombinant forms (URFs) were responsible for 4.4% (n = 14) infections.

### Complicated epidemic characteristics of CRF07_BC and CRF01_AE

**Figure 3:**
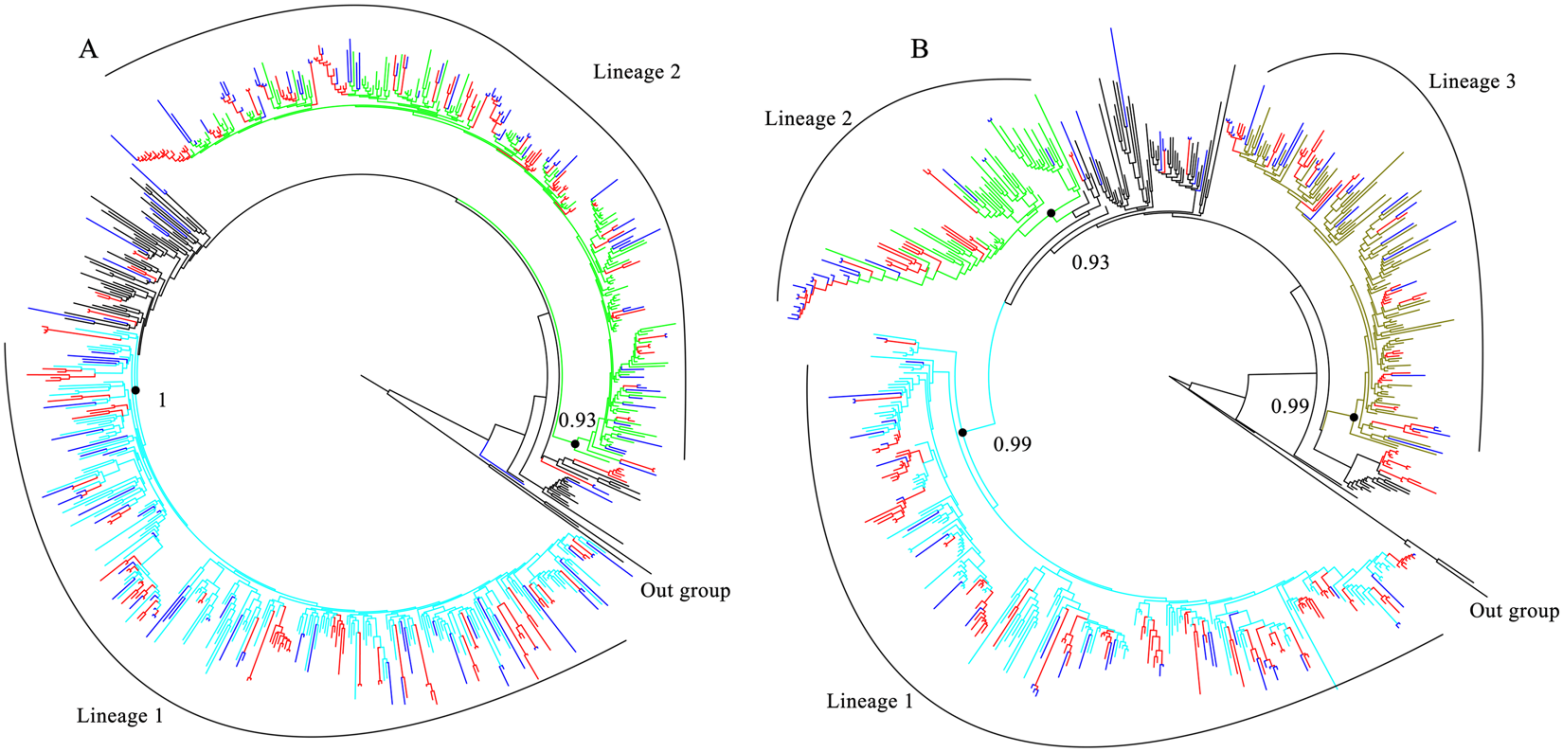
Phylogenetic trees of sequences with CRF07_BC (**A**) and CRF01_AE (**B**) infection. The phylogenetic tree was constructed using the approximately-maximum-likelihood method based on the *pol* region (HXB2: 2, 253 to 3, 312 nt) in FastTree 2.3. The nucleotide substitution mode was GTR + G + I. Bootstrap values ≥0.9 were used to identify lineages and were indicated at all relevant nodes. Genetic transmission clusters were defined by node support thresholds ≥95% and intra-cluster maximum pairwise genetic distances <3.0% nucleotide substitutions per site. Transmission clusters were depicted in red. Blue branches represented the sequences from Guangyuan. HIV-1 subtype J (**A**) and C (**B**) were chosen as an out-group in the rooted tree.

When compared with the demographic information between CRF07_BC and CRF01_AE infections, we found that CRF07_BC infections had a higher proportion of MSM (32.9% versus 23.8%), lower age (36.2 ± 13.3 years versus 42.8 ± 13.8 years), and higher of CD4+ T cell count (306.0, IQR: 201.0~402.0 cells/μl versus 254.0, IQR: 77.5~378.5 cells/μl) (Table 2). Phylogenetic analysis evidently identified two major distinct lineages (lineage 1–2) of CRF07_BC (Fig. 3A), involving in 90.1% (137/152) of Guangyuan city-related sequences. Three major distinct lineages (lineage 1–3) of CRF01_AE were identified (Fig. 3B), involving in 90.5% (95/105) Guangyuan city-related sequences. Notably, each lineage presented various epidemic characteristics implying a complexity of HIV-1 transmission among the subjects who ever went out as migrant workers in Guangyuan city (Supplementary Material 3).

**Table 2:** Distribution of the demographic information in various subtypes.

Variable	CRF07_BC n = 152 (%)	CRF01_AE n = 105 (%)	*P*
**Sex**			0.938
Male	121 (79.6)	84 (80.0)	
Female	31 (20.4)	21 (20.0)	
**Risk group** ^**a**^			0.032
M-HET	57 (37.5)	54 (51.4)	
F-HET	26 (17.1)	21 (20.0)	
MSM	50 (32.9)	25 (23.8)	
PWID/SU	19 (12.5)	5 (4.8)	
**Age (** $\bar{{\rm{x}}}$ ** ± s, years)**	36.2 ± 13.3	42.8±13.8	<0.001
**Marital Status**			0.771
Singlehood	66 (43.4)	45 (42.9)	
Married	74 (48.7)	54 (51.4)	
Unknown	12 (7.9)	6 (5.7)	
**Years of Education**			0.561
0–9	81 (53.3)	62 (59.0)	
≥10	60 (39.5)	38 (36.2)	
Unknown	11 (7.2)	5 (4.8)	
CD4+ T cell count (Median and IQR, cells/μl)	306.0 (201.0~402.0)	254.0 (77.5~378.5)	0.019
**Gone out as migrant worker before diagnosed** ^**b**^			0.667
Yes	103 (67.8)	67 (63.8)	
No	40 (26.3)	29 (27.6)	
Unknown	9 (5.9)	9 (8.6)	

^a^M-HET, male heterosexuals; F-HET, female heterosexuals; MSM, men who have sex with men; PWID, persons who inject drugs; SU, sexual transmission, unspecified type.

^b^The time of going out as migrant worker above 90 days before diagnosed.

### Identification of molecular transmission networks of HIV-1 in Guangyuan city

**Figure 4:**
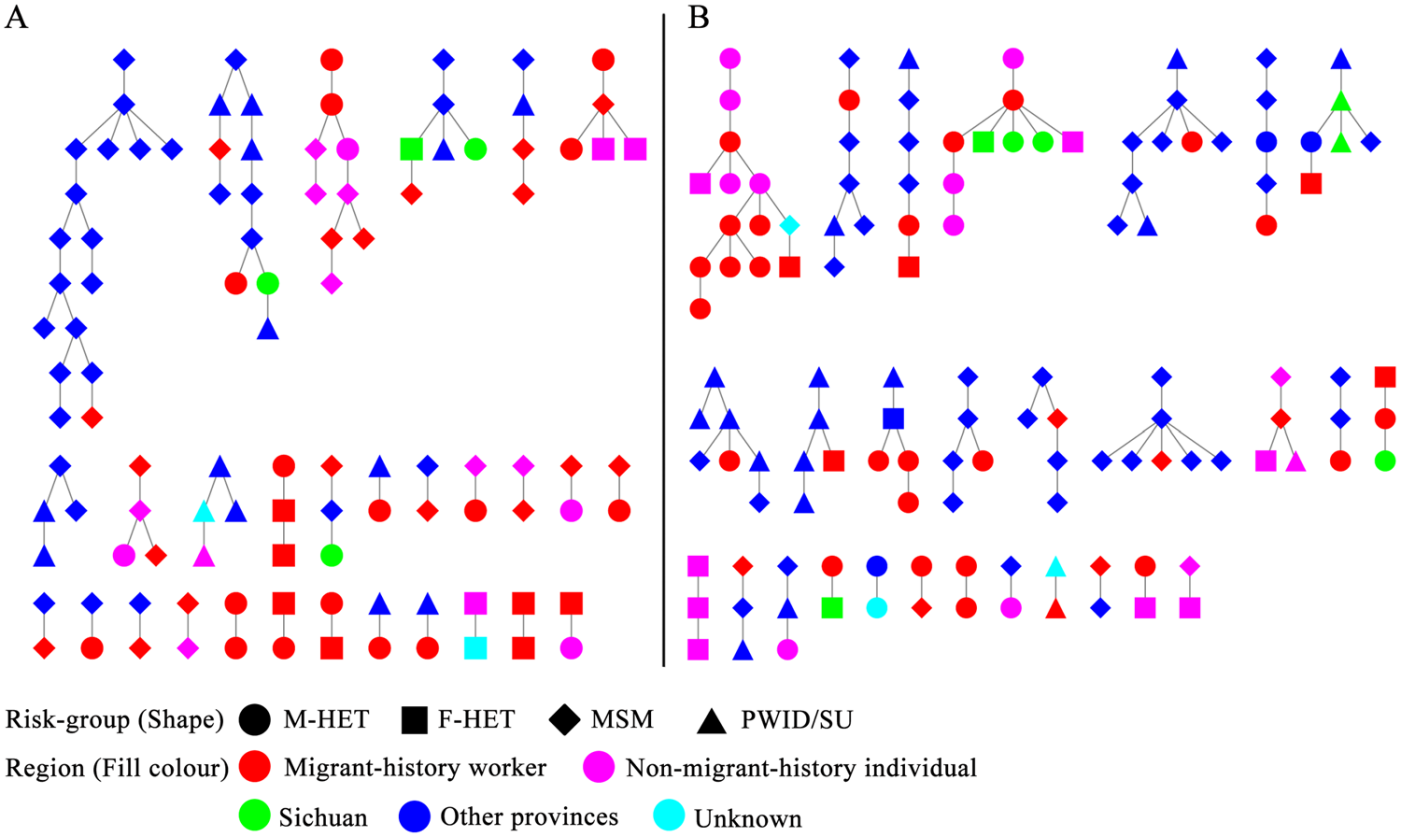
The transmission networks of CRF07_BC (**A**) and CRF01_AE (**B**) infected individuals. M-HET, male heterosexuals; F-HET, female heterosexuals; MSM, men who have sex with men; PWID, persons who inject drugs; SU, sexual transmission, unspecified type.

We focused on observing two major transmission networks, CRF07_BC and CRF01_AE. A total of 75 transmission clusters in the CRF07_BC networks were detected based on database_CRF07_BC_, where 59 Guangyuan city sequences were distributed in 29 Guangyuan city-related clusters (Fig. 4A). The clusters consisted of different sizes covering from 2 to 17 individuals, including 18 (62.1%), 7 (24.1%) and 4 (13.5%) for cluster size 2, 3–5 and >5 individuals, respectively. Of note, based on database_CRF01_AE_, a higher percentage of numbers in Guangyuan city-related sequences in the latter two cluster sizes were found in CRF01_AEnetwork than that in CRF07_BC (*P* = 0.024),where 9 (32.1%), 11 (39.3%), and 8 (28.6%) for cluster sizes 2, 3–5 and >5 individuals were respectively observed in 28 Guangyuan city-related clusters, (Fig. 4B). Interestingly, a large cluster existed in both CRF07_BC and CRF01_AE networks, with only Guangyuan city-related sequences (Fig. 4A,B). Both clusters had low mean genetic distance (0.00524 ± 0.00109 and 0.00421 ± 0.00062 nucleotide substitutions per sit), inferring a recent transmission of these two strains. The localCRF07_BC cluster included 9 male individuals, consisting of 6 MSM and 3 heterosexuals, among which 2 were diagnosed in 2012, 4 in 2014, 1 in 2015, and 2 in 2016, while theCRF01_AE cluster included 12 male and 2 female individuals, consisting of 1 MSM and 13 heterosexuals, among which each 6 were diagnosed in 2014 and 2015, respectively, and 2 in 2016.

### Migrant-history workers fueled HIV-1 transmission in Guangyuan city

**Figure 5:**
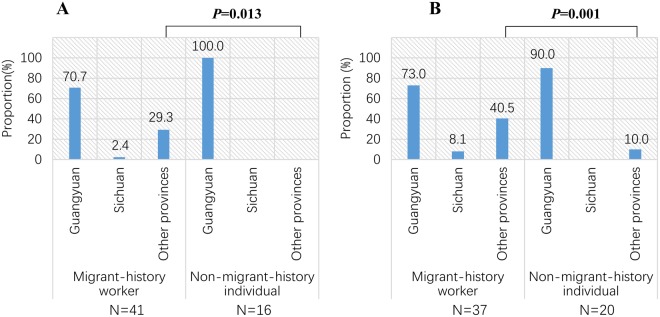
Links between Guangyuan city and other cities or provinces when stratified by migrant-history workers and non-migrant-history individuals in CRF07_BC (**A**) and CRF01_AE (**B**) networks. The column represented the proportion of migrant-history workers or non-migrant-history individuals linked with the Guangyuan, Sichuan and other provinces’ sequences, respectively, in networks.

Among all Guangyuan city sequence in CRF07_BC and CRF01_AE networks, the migrant-history workers were dominant. In CRF07_BC networks, there were 59 Guangyuan city sequence including 41 (69.5%) migrant-history workers and 16 (27.1%)non-migrant-history individuals. In CRF01_AE networks, there were 60 Guangyuan city sequence including 37 (61.7%) migrant-history workers and 20 (33.3%) subjects with no migrant history (non-migrant-history individuals). Interestingly, migrant-history workers linked more sequences from other provinces in both CRF07_BC and CRF01_AE networks (Fig. 5). Of 41 migrant-history workers in CRF07_BC networks, 29.3% (12) were linked to other provinces’ sequences. However, of 16 non-migrant-history individuals in CRF07_BC networks, no sequence was linked to other provinces’ sequence (*P* = 0.013, Fig. 5A). Similarly, of 37 migrant-history workers in CRF01_AE networks, 40.5% (15) were linked to other provinces’ sequences. But, only 2 (10%) subjects with no migrant history were linked to other provinces’ sequences in CRF01_AE networks (*P* = 0.001, Fig. 5B). While, by contrast, only 1 (2.4%) and 3 (8.1%) migrant-history workers linked with sequences from Sichuan in CRF07_BC and CRF01_AE networks, respectively.

Regarding to the HIV-1 transmission clusters between Guangyuan city and other provinces, over one fifth (20.3%, 12 of 59) of individuals were found within CFR07_BC network including Beijing (5.1%), Shanghai (5.1%), Guangdong (3.4%), Yunnan (3.4%), Xinjiang (1.7%) and Zhejiang (1.7%). Similarly, in CRF01_AE network, we found 28.3% (17 of 60) of individuals were involved the transmission between Guangyuan city and other provinces including Guangdong (10.0%), Shanghai (6.7%), Beijing (5%), as well as Fujian(1.7%), Hainan(1.7%), Henan(1.7%), Hunan(1.7%), and Shannxi(1.7%). (Table 3).

**Table 3:** Links between Guangyuan and other cities or provinces in the transmission networks.

Subtype	n	BJ	FJ	GD	HAN	HEN	HUN	SAX	SH	XJ	YN	ZJ	O/N
CRF07_BC	12	3 (5.1)		2 (3.4)					3 (5.1)	1 (1.7)	2 (3.4)	1 (1.7)	2 (3.4)
CRF01_AE	17	3 (5.0)	1 (1.7)	6 (10.0)	1 (1.7)	1 (1.7)	1 (1.7)	1 (1.7)	4 (6.7)				

GY, Guangyuan; SC, Sichuan (not including Guangyuan); BJ, Beijing; FJ, Fujian; GD, Guangdong; HAN, Hainan; HEN, Henan; HUN, Hunan; SAX, Shaanxi; SH, Shanghai; XJ, Xinjiang; YN, Yunnan; ZJ, Zhejiang; O/N, other/not available.

The Guangyuan city local linkages between different risk groups in the transmission networks were shown in Table 4. Of 26 Guangyuan city MSM in CRF07_BC networks, 80.8% (n = 21) were linked to other MSM, 26.9% (n = 7) to male heterosexuals, and 7.7% (n = 2) to female heterosexuals. Unexpectedly, of 20 Guangyuan city male heterosexuals, only 20.0% (n = 4) were linked to female heterosexuals, and on the contrary, a vast majority of them were linked to MSM and other male heterosexuals (75.0%, n = 15). Of 11 Guangyuan city female heterosexuals, 36.4% (n = 4) were linked to male heterosexuals, while 18.2% (n = 2) and 54.5% (n = 6) to MSM and other female heterosexuals, respectively. In CRF01_AE networks of 9 Guangyuan city MSM, 66.7% (n = 6) were linked to other MSM, 22.2% (n = 2) to male heterosexuals, and 33.3% (n = 3) to female heterosexuals. Unexpectedly, among the 35 Guangyuan city male heterosexuals, more than half of individuals (62.9%, n = 22) shared links with other male heterosexuals, while 25.7% (n = 9) were found to link to MSM and 22.9% (n = 8) to female heterosexuals. Of 13 Guangyuan city female heterosexuals, 46.2% (n = 6) were linked to male heterosexuals; however, 23.1% (n = 3) were linked to MSM and other female heterosexuals, respectively.

**Table 4:** Links between different risk groups in the transmission networks.

		n	MSM	Male HET	Female HET	O/N
CRF07_BC	MSM	26	21 (80.8)	7 (26.9)	2 (7.7)	2 (7.7)
	M-HET	20	10 (50.0)	5 (25.0)	4 (20.0)	3 (15.0)
	F-HET	11	2 (18.2)	4 (36.4)	6 (54.5)	
	PWID/SU	2				2 (100.0)
CRF01_AE	MSM	9	6 (66.7)	2 (22.2)	3 (33.3)	1 (11.1)
	M-HET	35	9 (25.7)	22 (62.9)	8 (22.9)	2 (5.7)
	F-HET	13	3 (23.1)	6 (46.2)	3 (23.1)	1 (7.7)
	PWID/SU	3	1 (33.3)			2 (66.7)

M-HET, male heterosexuals; F-HET, female heterosexuals; MSM, men who have sex with men; PWID, persons who inject drugs; SU, sexual transmission, unspecified type.

## Discussion

As the uneven economic development in China, massive migrant-workers traveled from the rural areas to urban centers to seek better employment opportunities and to improve their living conditions. By the end of 2017, the size of outgoing migrant-workers had reached 171.9 million nationwide, and the majority of them were living in some international metropolis, such as Beijing, Shanghai, Guangzhou, *et al*.^10^. The strong association between HIV-1 infection and migrant-workers was well-established^16–18^. Engaging in high-risk behaviors^5^ made them more vulnerable to HIV-1 than the general population^19^. In addition, the high mobility of migrant-workers makes it difficult to monitor HIV-1 infection and manage care^20^. Guangyuan city in Sichuan was well-known as a labor-exporting city with approximately one million migrant-workers every year^21^. Overall, we elucidated the characteristics of HIV-1 genetic diversity, viral evolutional lineages, and transmission networks in this city. Two distinguishing features of HIV-1 epidemics in Guangyuan city included: 1) there were close transmission linkages between Guangyuan city and other provinces or municipalities, especially some international metropolis, for which people who ever went out as migrant-workers probably played a crucial role; 2) the vast majority of male heterosexuals were observed being shared links with other male heterosexuals and MSM, implying that some MSM might hide their real sexual orientation.

In this study, we found a remarkably high portion of a migrant-history HIV reported cases in Guangyuan city, and most of the migrant-history workers were outside the home province. The studies in some single towns (e.g., Unite Kingdom and Uganda), showed that only small amount of the transmission events occurred locally^22–24^. To further identify the reliable linkages between HIV reported cases in Guangyuan city and other areas, a wide range of reference sequences were included for both phylogenetic analysis and transmission network analysis. As expected, our results revealed that Guangyuan city sequences had a close link with sequences form other cities. Furthermore, a much higher proportion of links were found with sequences derived from the Yangtze, Zhujiang River delta areas and the Bohai Rim than that from other cities of Sichuan. Based on the statistical data, an approximately above 50% migrant-workers of Guangyuan city constantly flow to the cities of these areas, such as Guangdong (106.9 thousand), Zhejiang (86.3 thousand), Shanghai and Jiangsu (62.7 thousand), as well as Beijing and Tianjin (61.1 thousand)^9^. Thus we inferred that the migrant-workers flow between their work place and home with not only money but also HIV-1, for their higher levels of sexual risk including unprotected sex^25^. And they might serve as a bridge and facilitated viral transmission from other provinces/cities to home regions.

Although heterosexual contact remained the domination of acquiring the HIV-1 infection in Guangyuan city, the proportions of MSM in newly diagnosed HIV-1 infections posed a persistent increase in recent years. Usually, urban areas had more attraction to MSM, since large cities had relatively open culture and convenient sexual venues (bars, saunas, parks, sex clubs, *et al*.)^18^. These MSM migrants were more at highest risk for HIV-1 than other individuals in the migrant-workers^26–28^. More disturbing, a number of MSM might hide their real sexual orientation in China, because of the traditional cultural, historical reasons, as well as societal stigma and discrimination^29^, which could be wildly underestimating the role of the MSM in the HIV-1 transmission networks. Especially, the frequent high-risk bisexuality and unprotected behaviors of these MSM make their female partners more vulnerable to HIV-1^30,31^. This also suggested that the real infected routes of these male heterosexuals in the large cluster deserved further investigation. Importantly, the low mean genetic distance in two lager clusters with recently diagnosed individuals in both CRF07_BC and CRF01_AE networks illustrated that the dynamic networks would probably continue to expand in the future if not intervened.

However, the incomplete coverage of sequences might make our results subject to selection/sampling bias. We anticipated being able to make even stronger inferences about the transmission network characteristic through improving the completeness of molecular surveillance data in the future.

## Conclusions

We, for the first time, revealed the current HIV-1 epidemic in a hotspot area of Sichuan province that exported labors through analysis of viral diversity and transmission networks, and further underscored the value of molecular sequences survey combined with epidemiologic and demographic data in guiding precautionary intervention at the local level.

## Electronic supplementary material

Supplementary Material
