# Supplementary material for: Impact of worker emigration on HIV epidemics in labour export areas: a molecular epidemiology investigation in Guangyuan, China
Source: Sci Rep. 2018 Oct 30;8:16046. doi: 10.1038/s41598-018-33996-6 (PMC6207672; doi:10.1038/s41598-018-33996-6)
Supplement: Supplementary file 1 — Supplementary Material [file 41598_2018_33996_MOESM1_ESM.docx]

**Impact of worker emigration on HIV epidemics in labour export areas: a molecular epidemiology investigation in Guangyuan, China**

Ling Su^a^, Shu Liang^a^, Xueqin Hou^b^, Ping Zhong^c^, Dongbin Wei^a^, Yu Fu^b^, Li Ye^a^, Li Xiong^b^, Yali Zeng^a^, Ying Hu^a^, Hong Yang^a^, Bo Wu^d^, Linglin Zhang^a*^,Xiaoshan Li^d*^

^a^Center for AIDS/STD Control and Prevention, Sichuan Provincial Center for Disease Control and Prevention, Sichuan, China

^b^Center for AIDS/STD Control and Prevention, Guangyuan Municipal Center for Disease Control and Prevention, Gunagyuan, China

^c^Department of AIDS and STD, Shanghai Municipal Center for Disease Control and Prevention, Shanghai Municipal Institutes for Preventive Medicine, Shanghai, China

^d^Department of Lung Transplant Center, Affiliated Wuxi People’s Hospital of Nanjing medical University, Wuxi, Jiangsu, China

**Running title:** Impact of worker emigration on HIV epidemics in labour export areas

^*^Correspondence to:

Xiaoshan Li, MD

Department of Lung Transplant Center, Affiliated Wuxi People’s Hospital of Nanjing medical University, Wuxi, Jiangsu, China, 299 Qingyanglu Road (W), Wuxi 214023, China

Tel: 86-0510-85350996Fax: 86-0510-85350996

Email: [sclxs87@163.com](mailto:sclxs87@163.com)

Linglin Zhang

Sichuan Provincial Center for Disease Control and Prevention, Center for AIDS/STD Control and Prevention, Sichuan, China. Wuhou district Zhongxue road 6#, Chengdu 610041, China

Tel: 86-028-85586957

Email: zhangllsccdc@163.com

**Supplementary material 1. The number of newly diagnosed HIV-1 in Guangyuan every year and the number of *pol* sequences included in this study.**

| Time | Before 2012 | 2012 | 2013 | 2014 | 2015 | 2016 | 2017 | Total |
| --- | --- | --- | --- | --- | --- | --- | --- | --- |
| The number of newly diagnosed HIV-1 | 163 | 81 | 89 | 135 | 143 | 171 | 32 | 814 |
| The number of *pol* sequences included in this study | 0 | 49 | 49 | 79 | 66 | 54 | 18 | 315 |
| The sampling ratio (numbers of *pol* sequences over number of newly diagnosed HIV-1), % | 0.0 | 60.5 | 55.1 | 58.5 | 46.2 | 31.6 | 56.3 | 38.7 |

The *pol* sequences were collected from January 1, 2012 to February 28, 2017.

**Supplementary material 2. The distribution of patristic genetic distance of all links and Guangyuan-related links in CRF07_BC (A) and CRF01_AE (B) networks.** The median genetic distance for all links and Guangyuan-related links in CRF07_BC networks were 0.00522 (IQR: 0.00284~0.00957) and 0.00738 (IQR: 0.00284~0.01057) nt substitutions per site, respectively. The median genetic distance for all links and Guangyuan-related links in CRF01_AE networks were 0.00552 (IQR: 0.00339~0.00857) and 0.00455 (IQR: 0.00314~0.00800) nt substitutions per site, respectively.


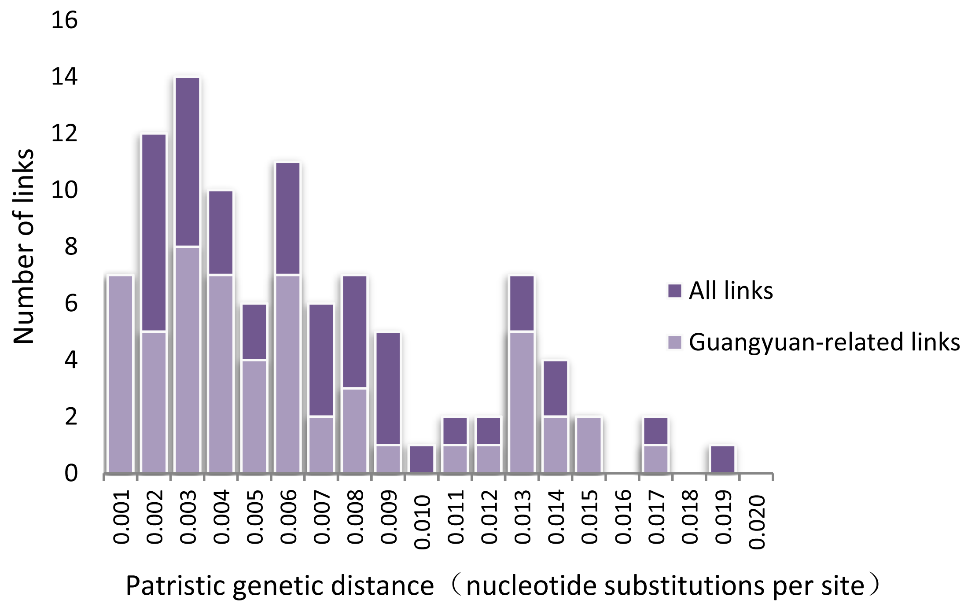


(A)


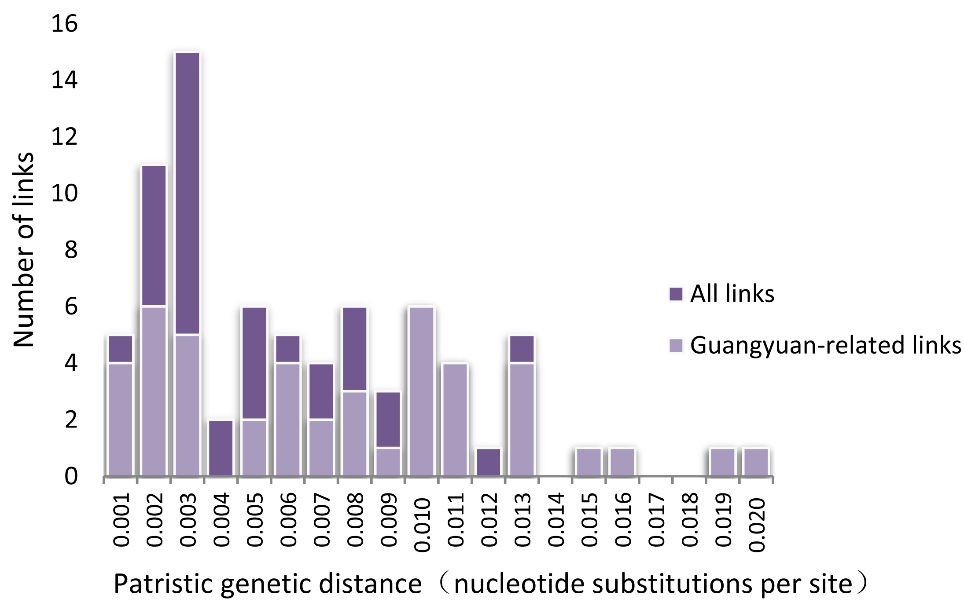


(B)

**Supplementary material 3. Distribution of the demographic information in various lineages.**

| **Variable** | **CRF07_BC** | |  | **CRF01_AE** | | |
| --- | --- | --- | --- | --- | --- | --- |
|  | **Lineage 1**  **58 (%)** | **Lineage 2**  **79 (%)** |  | **Lineage 1**  **39 (%)** | **Lineage 2**  **26 (%)** | **Lineage 3**  **30 (%)** |
| **Sex** |  |  |  |  |  |  |
| Male | 36 (62.1) | 75 (94.9) |  | 33 (84.6) | 20 (76.9) | 23 (76.7) |
| Female | 22 (37.9) | 4 (5.1) |  | 6 (15.4) | 6 (23.1) | 7 (23.3) |
| **Risk group ^a^** |  |  |  |  |  |  |
| M-HET | 23 (39.6) | 28 (35.4) |  | 10 (25.6) | 18 (69.2) | 20 (66.7) |
| F-HET | 19 (32.8) | 3 (3.8) |  | 6 (15.4) | 6 (23.1) | 7 (23.3) |
| MSM | 4 (6.9) | 44 (55.7) |  | 19 (48.7) | 2 (7.7) | 2 (6.7) |
| PWID/SU | 12 (20.7) | 4 (5.1) |  | 4 (10.3) | 0 (12.8) | 1 (3.3) |
| **Age (**$\bar{\mathbf{x}}$**±s, years)** | 40.1±14.9 | 32.3±9.9 |  | 36.8±12.6 | 48.7±10.5 | 48.0±14.0 |
| **Marital Status** |  |  |  |  |  |  |
| Singlehood | 16 (27.6) | 49 (62.0) |  | 26(66.7) | 7(26.9) | 8(26.7) |
| Married | 35 (60.3) | 26 (32.9) |  | 12(30.8) | 18(69.3) | 18(60.0) |
| Unknown | 7 (12.1) | 4 (5.1) |  | 1 (2.5) | 1 (3.8) | 4 (13.3) |
| **Years of Education** |  |  |  |  |  |  |
| 0-9 | 40 (69.0) | 29(36.7) |  | 22(56.4) | 21(80.8) | 19(63.3) |
| ≥10 | 12 (20.7) | 46(58.2) |  | 16(41.1) | 4(15.4) | 8(26.7) |
| Unknown | 6 (10.3) | 4 (5.1) |  | 1 (2.5) | 1 (3.8) | 3 (10.0) |
| **CD4+ T cell count (Median and IQR, cells/μl)** | 278.0  (142.5~399.5) | 324.0  (240.0~418.0) |  | 239.0  (13.0~377.0) | 264.0  (122.0~340.5) | 273.0  (159.3~402.8) |
| **Gone out as migrant worker before diagnosed ^b^** |  |  |  |  |  |  |
| Yes | 38(65.5) | 56(70.9) |  | 25(64.1) | 14(53.9) | 22(73.3) |
| No | 16(27.6) | 22(27.8) |  | 11(28.2) | 11(42.3) | 5(16.7) |
| Unknown | 4 (6.9) | 1 (1.3) |  | 3 (7.7) | 1 (3.8) | 3 (10.0) |

^a^ M-HET, male heterosexuals; F-HET, female heterosexuals; MSM, men who have sex with men; PWID, persons who inject drugs; SU, sexual transmission, unspecified type.

^b^The time of going out as migrant worker above 90 days before diagnosed.
